# An Exposition of Quackery and Imposture in Medicine, &c.

**Published:** 1839-04-01

**Authors:** 


### AN EXPOSITION OF QUACKERY AND IMPOSTURE IN MEDICINE, &c. By Dr. *Ticknor*, with notes by *W. Wright*, Surgeon-Aurist. S. Hodson, Fleet-street.

"QUACKERY and imposture" will continue in medicine, so long as there are people to be gulled, and lucre obtained by gulling. This "we calculate" will be for ever. Is quackery or imposture confined to physic? No verily. It has existed in every profession and trade from the very origin of arts, and is only now more extensive than hitherto, because *Arts* are more numerous and general. Was smuggling ever put down by penal enactments? Never. It can only be annihilated by rendering the trade unprofitable—by taking off the duties. Can quackery and imposture ever be rendered unprofitable in physic, or in any profession? We think not. It exists and has always existed even in religion. The priests were quacks and impostors from the earliest ages, when they pretended to have influence on the skies, and to even hold the keys of Heaven. Have we not plenty of quacks and impostors in religion at this very day! How often is the mask of sanctity worn by the hypocrite for the purpose of obtaining his own selfish ends? Do not the lawyers *impose* on their credulous clients and urge them to suits where the lawyer only is the winner. Is there a single lawsuit in which one party is not deceived by the opinions of their legal advisers? But there is not a single art, profession, or science into which "quackery and imposture" do not force their way—and medicine cannot expect to escape. This science, if indeed it be one, is more open to quackery and imposture than any other. One half of our practice is guess-work, and in matters of such uncertainty the ignorant will have his guess, and assert that he knows more than his neighbours. But then there are *unqualified* men who practise physic without a diploma—and consequently are quacks. This is a very small portion of the evils of quackery and imposture in medicine! The mass of trickery and humbug in medicine—and legalized too—is to *unlicensed* practice as the Atlantic ocean to the lake of Keswick. What is the unbounded practice of detraction but quackery and imposture! A medical man is shewn the prescription of another medical man, and, as a matter of course, pronounces it to be totally inappropriate to the case, significantly hinting his astonishment that the patient is alive to tell his tale! Is this practice frequent? More frequent, we believe, than unlicensed practice—and it is more disgraceful—more unchristian—and more detrimental (si sua bona norint) in the end to the medical profession. Many, we have no doubt, commence this *mala praxis* from necessity, and continue it from *habit,* without sinister intentions —as liars at last believe their own falsehoods, so often repeated.

The object of the work before us is to "overthrow quackery in medicine, by exposing its errors, &c." Good intentions, no doubt, but quite impracticable. Dr. Ticknor tells us that—

"It is neither the ignorant nor the superstitious alone who are gulled by the designing; for in this country such individuals are rarely to be found—and yet there are plenty of subjects upon whom these harpies prey." 6.

Thus then amongst the learned and intelligent, in the United States, as indeed in this country, the quack finds his ready customer! And what is the remedy proposed by our author? A general study of medicine by the community. The thing is impossible—and if it could be put into execution, the mischief would be incalculable. The smattering of medical science which could possibly be attained by the masses, would only render one half of them hypochondriacs—and consequently the prey of charlatans. Even medical men themselves are far more frightened by their own ailments than are the most ignorant of their patients. But how would it be possible for one in one thousand of the community, to acquire even a smattering of anatomy, chemistry, *practice* of medicine, &c. The thing is as Utopian as any scheme ever propounded to the world.

The greater portion of the work consists of a slight popular delineation of health, disease, and remedies—interlarded, however, with a great many anecdotes of quackery and its victims. These are, of course, the only portions of the book that can claim any notice from the medical reader—and, to say the truth, they are the portions of the work which will do most good to the community—by drawing their attention to the iniquities of empiricism —and the gullibility of its customers. We shall cull a few samples from these anecdotes.

"An empiric of the first water, not many years ago, had made himself famous for the cure of all human maladies, by the administration of peculiarly large pills of his own invention. What contributed not a little to the increase and spread of his reputation was the fact, that he used frequently to tell his patients, that from their symptoms he was confident some particular substances were lodged in a portion of the alimentary canal. At one time he would tell a patient that he had apple seeds retained in his bowels; and again he would tell another, that he had kernels of different fruits, and grains, in his stomach, and if by questioning gentlemen he could ascertain that they were fond of shooting, it was not seldom that he attributed their complaints to having accidentally swallowed a few shot. As nothing could so conclusively prove his prognostics correct, as the simple fact of finding the articles named, so the old gentleman's character for wisdom and skill became more and more firmly established; for the identical causes of mischief were invariably discovered after taking a dose of the 'big pills.' At length, a lady of the first respectability, having suffered a long time from deranged digestion, applied to the celebrated doctor for assistance. After a few questions, he told her very promptly that he understood her complaint, that he knew what ailed her, and more than all, that her doctor was a fool, and assured her that his big pills would effect a cure. Neither of these assertions she exactly credited, but nevertheless, concluded, to try his remedy if he would make known to her the complaint. 'Why,' says he, 'you have got lemon seeds in you—you must take some of my big pills and get rid of them, and you'll be perfectly well again.' 'Why, Doctor,' said the lady in amazement, 'I have not eaten a lemon for six years; and what you say is altogether impossible.'

'No matter, madam, if you have not eaten a lemon for twenty years, the fact is just as I tell you, and if you will take the pills you can be satisfied of it.'

The pills were taken, and to the utter astonishment of the patient, the lemon seeds were found; a second dose was taken and still more seeds made their appearance. A thought now flashed upon the lady's mind. One pill was yet left which she examined, and behold! *a lemon seed* in its centre—the secret, truly, of the Doctor's astonishing wisdom, and successful practice." 49.

This was no bad trick of the transatlantic charlatan, and we should not be at all surprized to find the hint taken by some cisatlantic worthies of the same class.

2. It appears that the lobelia inflata is working its mischiefs on both sides of the Western Ocean. We observe, almost every day, in this Metropolis, the injuries occasioned by the administration of the tincture, in what are called asthmas, but which are, in reality, neither more nor less than bronchial inflammations. In such cases, as might be expected, the irritating tincture of lobelia inflata does much harm.

"'A melancholy instance of death occasioned by the use of this plant in the hands of a quack, is detailed in the sixth volume of the Massachusetts Reports, in the trial of Samuel Thompson, an empiric, practising in Beverly, for the murder of Ezra Lovett. In this trial, it appeared that the patient, being confined by a cold, sent for the pretended physician, who gave him three powders of lobelia in the course of half an hour, each of which vomited him violently, and left him in a great perspiration during the night. The next day two more powders were administered, each of which operated by vomiting, and occasioned great distress. In like manner two other powders were given the subsequent day, leaving the patient in a state of great prostration. Several days after this the physician (?) came again, and finding his patient still worse, administered several more powders, which occasioned great distress, and at length ceased to operate. Finding that the stomach was not sensible to the emetic effect of the lobelia, the physician (?) repeated the dose, and when the patient complained of great distress at the breast, and said he was dying, the doctor (?) assured him the medicine would soon get down, or operate as a cathartic. However, on the same evening, the patient lost his reason, and became convulsed, so that two men were required to hold him. To relieve which, the doctor forced down two more of his powders, and the patient, as was expected, grew worse, and continued so until he expired.

'The doctor, who had thus terminated the disease and the patient at once, was arrested and put upon trial for murder. But the homicide proving a legitimate one, from the want of a sufficient evidence of malice prepense, he was acquitted and set at liberty.'" 56.

This was what the lawyers would call "sharp practice"—even for brother Jonathan!

On the shores of liberty and equality they have a new species of physicians —"*the Steam Doctors.*" These are not steam-boat doctors—but itinerant medici who go about the country with portable vapour-baths, curing all diseases by steam and cayenne pepper! These doctors compress the whole circle of medical science into a duodecimo volume,—the direction for steaming, and with this pandect each one sallies forth, like a Knight errant, to relieve all damsels, distressed widows, and sick children.

"Like a harpy, he scents his prey at a distance; and wherever he can hear of a sick person, no matter whether he is attended by a physician or not, thither he directs his course; and by impudence, promises of a certain cure, and denunciations of 'apothecary doctors,' attempts to impose upon the patient, and his friends, his homicidal quackery. This is not all, for sometimes he is successful in perpetrating his practice upon the credulous; and happy is the patient who lives to boast of his recovery.

A steaming quack, of a stamp like the one above described, made an effort to introduce the steam and red pepper practice into the family of his friend, who had four children lying sick with scarlet fever, of a most inflammatory character." 70.

With one more extract we shall conclude. It may be perused with advantage by those who raise a cry in this country in favour of cheap medical education—and against long courses of study, with their attendant expenses.

"It is the peculiar excellence of our government, and our pride and boast, that all who live under it are equal, in point of privilege, and in respect to person. 'Liberty and equality,' 'democracy and republicanism,' are the watchwords of the day, and on these foundations, thank Heaven, our institutions are reared; but there is a limit beyond which liberty degenerates into licentiousness and when democracy is but another name for the rule, or rather misrule, of the rabble. It is not patriotism, or love to our fellow-men which would confer the same privileges on the learned and on the ignorant—on the virtuous and on the vicious—it is not republicanism which would lower the standard of the professions, and open the portals of science for all to enter, whether qualified or not. Instead of lengthening the term of study or requiring more qualifications of a candidate for graduation, a cry of 'aristocracy' is at once raised—the poor cry, 'persecution;' they conceive their rights invaded, and an effort is made to produce a generation of doctors, of a character inferior to all their predecessors. It is a fact that wealth always does, and ever will confer power; in commerce, agriculture, manufactures, trade and speculation, capital is necessary. Those who have it not find no fault with those who possess it, because they see that none of these operations can be carried on without it. But, talk of matters of science, and the necessity of capital—of the expenditure of time and money to aid in its acquisitions—and the cry is, by those in and out of the profession, 'that will never do, it costs too much already to learn to be a doctor; you must bring the professions within our reach; let the man of three years' superficial reading enjoy all the honours and privileges of one who has devoted ten times the amount of labour to the same object; you must, in fact, degrade science to suit our circumstances, and our convenience'—this is pure 'democracy,' this is 'equality and republicanism.'" 232.

We wish rather than hope that Dr. Ticknor's volume may achieve the object for which it was written—the annihilation of quackery. If it perform this task, it will outstrip any of the labours of Hercules—or all twelve put together. And when it suppresses all *unlicensed* quacks, the Doctor will have plenty of *unqualified* practitioners to sweep from society—and even when all are brought up in learning, to high-water mark, his labours are only beginning: he will then have the unbounded field of *legitimate* quackery and imposture to weed. He will have the homœopathic humbugs—the monomaniac magnetisers—the lung-stretching "consumption-curers"—and ten thousand other heads of the hydra of quackery to combat—in which contest the worthy Doctor will be beaten out of the field, and laughed at for his pains.

